# Assessing the credibility of COVID-19 vaccine mis/disinformation in online discussion

**DOI:** 10.1177/01655515211040653

**Published:** 2021-08-19

**Authors:** Reijo Savolainen

**Affiliations:** Faculty of Information Technology and Communication Sciences, Tampere University, Finland

**Keywords:** COVID-19 vaccines, credibility, disinformation, misinformation, online discussion

## Abstract

This study examines how the credibility of the content of mis- or disinformation, as well as the believability of authors creating such information is assessed in online discussion. More specifically, the investigation was focused on the credibility of mis- or disinformation about COVID-19 vaccines. To this end, a sample of 1887 messages posted to a Reddit discussion group was scrutinised by means of qualitative content analysis. The findings indicate that in the assessment of the author’s credibility, the most important criteria are his or her reputation, expertise and honesty in argumentation. In the judgement of the credibility of the content of mis/disinformation, objectivity of information and plausibility of arguments are highly important. The findings highlight that in the assessment of the credibility of mis/disinformation, the author’s qualities such as poor reputation, incompetency and dishonesty are particularly significant because they trigger expectancies about how the information content created by the author is judged.

## 1. Introduction

Many of the recent investigations on information credibility focus on the user-generated data available in social media platforms [1–3]. The growing interest in credibility issues is mainly due to the proliferation of mis- and disinformation, ranging from fake news to false rumours about the COVID-19 pandemic. From this perspective, COVID-19 is a particularly interesting case because there are still many unanswered questions about the disease and its effective prevention, making it easy for rumours to take root in the absence of scientific certainty. For example, anti-vaccination activists have occupied a visible role in social media forums by amplifying distrust of COVID-19 vaccines and spreading disinformation about their nature and side effects [4]. The discourse on COVID-19 vaccination reflects the ‘post-truth’ era which is characterised – as Gibson and Jacobson [5] put it – by the ‘swirling cacophony of competing viewpoints, perspectives, agendas, and facts’. The contestation of the expertise of medical professionals offers a fertile ground for the growth of scepticism and reinforces ‘folk wisdom’ about vaccination in the social media forums [5].

The present investigation contributes to empirical research on information credibility by examining a timely issue: how is the credibility of anti-vaccination claims assessed in online discussion? The study offers a novel viewpoint on this topic by approaching anti-vaccination claims as potential carriers of mis- or disinformation. *Misinformation* refers to false and inaccurate (vague and/or ambiguous) information that is communicated regardless of an intention to deceive, while *disinformation* denotes false (deceptive) information that is spread knowingly and deliberatively [6]. If a person genuinely believes, for example, that a COVID-19 vaccine as a side effect causes autism, we cannot challenge the existence of his or her belief. If such a belief is shared in an online discussion forum, people advocating the safety of COVID-19 vaccines may assert that the above person is distributing misinformation. However, if the person knows that a COVID-19 vaccine is unlikely to cause autism but still deliberately spreads false rumours about the connection between the vaccine and the disease, he or she is distributing disinformation about a COVID vaccine.

So far, however, we lack more detailed knowledge about how and by which criteria people assess the credibility or non-credibility of vaccine mis- or disinformation in online discussion. This is an important issue particularly in situations in which people hesitant about COVID-19 vaccines make decision whether or not to get vaccination, based on the wealth of objective and factual, as well as opinionated mis/disinformation available in social media forums. To bridge this gap, an empirical study was made by analysing the messages posted to a Reddit online discussion group. More specifically, the analysis was focused on the ways in which so-called ‘pro-vaxxers’– people advocating vaccination – criticise COVID vaccine mis- and disinformation distributed by ‘anti-vaxxers’, that is, people advocating anti-vaccination. The Reddit discussion group was chosen for the study because the critiques presented by the pro-vaxxers incorporate explicit judgements of the credibility of vaccine information spread by the anti-vaxxers. As this study concentrates on the credibility assessments, the question of whether an anti-vaxxer in a certain message distributes mis- or disinformation is secondary. Therefore, no attempts will be made to distinguish between the above forms of information; they are simply referred to as *mis/disinformation*. As a major contribution, the empirical findings elaborate the picture of information credibility by demonstrating how people assess the credibility of authors distributing mis/disinformation, as well as the credibility of the content of such information.

The rest of the article is organised as follows. In section 2, the main approaches to credibility assessment are characterised, followed by the review of vaccine hesitancy as a public health issue and source of mis/disinformation. In sections 3 and 4, the conceptual framework, empirical research questions and research methodology are specified. The empirical findings are reported in section 5. Sections 6 and 7 discuss the research findings and draw conclusions on their significance.

## 2. Literature review

### 2.1. Approaches to the assessment of information credibility

Despite the long-time research in diverse fields such as philosophy, communication studies and information science, there is no universal agreement on what dimensions constitute credibility. Researchers have approached it in diverse terms such as believability, trust, reliability, accuracy and objectivity [7,8]. The exact definition of the construct of credibility is also rendered more difficult because it is closely related to the concept of *quality* which similar to credibility is multifaceted in nature. The conceptual ambiguity is reflected in that information science researchers often use the term quality to denote the concept of credibility [9]. However, the category of credibility may be used to denote the aspects of information quality. Moreover, credibility and quality judgements may occur together when an individual assesses the reliability of a message [10]. Despite this contingency, researchers have made attempts to distinguish between information quality and information credibility. For example, Rieh [11] specified information quality as ‘a user criterion which has to do with excellence or in some cases truthfulness in labeling’. More specifically, at an operational level, information quality was identified as ‘the extent to which users think that the information is useful, good, current, and accurate’ (p. 146) [11]. However, the exact operationalisation of the above construct is difficult because the aspects of information quality are not necessarily consistent. For example, a fact such as yesterday’s temperature at 12 p.m. in London may be accurate, but no longer useful for an individual; however, a fact can be current but inaccurate (e.g. the number of today’s COVID-19 cases in India). Thus, there is often a need to support the judgement of information quality by assessing the credibility of information. The individual judging the quality of information such the number of COVID-19 incidents in India has to consider whether the source of information, for example, a website can be taken seriously. From this perspective, the judgement of information quality and credibility is closely related. For example, Rieh and Danielson [9] suggested that credibility is a principal component of information quality. Savolainen [12] proposed that information quality is primarily be related to the content of a message, for example, its currency, factuality, reliability and specificity. To compare, information credibility is mainly dealing with the attributes of the author of the message, more precisely, his or her expertise, as well as his or her ability to provide evidence to support the believability of information.

The above characterisations suggest that similar to information quality, information credibility can be defined as a concept in its own right. To achieve this, researchers have often approached credibility in terms of believability, trust, reliability, accuracy, fairness and objectivity [7]. Rieh [13] contends trustworthiness is a core dimension in credibility because it captures the perceived goodness and morality of the source. A person is trustworthy for being honest, careful in choice of words and disinclined to deceive [7]. Information is trustworthy when it appears to be reliable, unbiased and fair. Despite the variety of approaches to the concept of credibility, most researchers agree, however, that the key dimensions of credibility are *trustworthiness* and *expertise*. Information is trustworthy when it appears to be reliable and unbiased. Expertise indicates an individual’s ability to provide information that is both accurate and valid [7]. Researchers have also distinguished between message and source credibility. *Message credibility* indicates how message characteristics impact perceptions of believability [8]. Of such characteristics, message content is particularly important since it indicates the extent to which information available in the message is correct and accurate. *Source credibility* is indicative of the believability of the author of the message in terms of his or her perceived reputation, expertise and honesty.

Importantly, perceptions of credibility may differ depending upon the type of source being evaluated and the context in which the evaluation occurs. For example, commercial information tends to have low credibility; people tend to discount information from sources with obvious persuasive intent [14]. Moreover, the familiarity with the site genre as a source of a particular kind of information (which perhaps triggers particular pre-message expectancies) is an important component of credibility perceptions [14]. Pre-message expectancies are based on the fact that information type can signal a relative persuasive intent, thus eliciting in the user a corresponding level of trust or scepticism they might bring to bear on source, message or site credibility. This is also evident in the context of this study, that is, Reddit online discussion forum critiquing the ideas of anti-vaccination. From the outset, such ideas are perceived as something dubious from the perspective of the pro-vaccination participants. Therefore, due to pre-message expectancies, messages that exist in an online context where explicit persuasive intent may be present are subject to lower credibility assessments. This may be due to a higher scrutiny or scepticism. The online participants expect that anti-vaccination ideas are low in credibility, presumably because they are sceptical about the intentions of authors submitting opinionated messages, as well as the veracity of online information of this kind. In this regard, the perceived honesty of the author is particularly important for the credibility judgement [12]. To this end, the participants consider whether the author is able to consider an issue in a sincere way and whether or not information presented in his or her messages could be taken seriously.

There are a growing number of empirical investigations examining credibility assessments made in the social media forums. Kim [15] analysed how the questioners assessed the credibility of answers presented in Yahoo! Answers – a social question and answer (Q&A) site. While judging message credibility, the questioners most frequently drew attention to the logic or plausibility of the arguments presented by the answerer. Moreover, message criteria such as accuracy, clarity, layout, spelling/grammar and tone of writing were important. In the assessment of source credibility, the most frequently mentioned criteria included the answerers’ perceived expertise, honesty and reference to external sources. Savolainen [12] examined how online discussion participants judge the quality and credibility of information dealing with two controversial topics: the usefulness of natural products (or health food) and racism. Information quality was analysed by focusing on the features of the message while information credibility was examined by concentrating on the characteristics of the author of the message. In the evaluation of information quality, the most frequently used criteria pertained to the usefulness, correctness and specificity of information. In the judgement of information credibility, the author’s reputation, expertise and honesty appeared to be a particularly important. Overall, Savolainen’s [12] findings are characteristic of credibility assessments made in the context of sensitive discussion topics because negative judgements indicating distrust in information quality and information credibility were highly frequent.

More recently, Lee and Shin [16] demonstrated that one of the factors affecting the credibility assessment is the extent to which people are aware of whether the material available in social media platforms represents truthful (objective) information or mis/disinformation. For example, health-related mis/disinformation may be found useful and credible because people tend to seek evidence that corroborates their existing beliefs [16]. It is also possible that the mere presence of pseudo-evidence boosts perceived credibility of misinformation among audiences who are neither particularly motivated nor able to critically evaluate the information. Nonprobative information such as photos or verbal descriptions may produce ‘truth bias’, significantly inflating subjective feelings of truth [16]. Moreover, the credibility of mis/disinformation may be strengthened by repeating claims about an issue, for example, the inherent risks of COVID-19 vaccines. The more often people encounter such claims, the more probably they are to find the claims to be true.

### 2.2. Vaccine hesitancy as a public health issues and source of mis/disinformation

Since the 1800s, vaccines have had a large impact on health worldwide, resulting in the eradication of diseases such as smallpox and preventing millions of deaths annually [17] However, there is a long tradition of *vaccine hesitancy* which refers to a delay in acceptance or refusal of vaccines, despite availability of vaccination services [18]. The fear of vaccination risks is not irrational because there are examples of vaccination disasters. A contaminated batch of polio vaccine caused the paralysis or death of 56 children in 1955 [19]. In 2009–2010, the Pandemrix vaccine against swine flu caused a controversy in many countries, due to its association with the incidence of narcolepsy [20]. This may have exacerbated vaccine hesitancy, especially related to influenza vaccines, as well as the vaccines against COVID-19.

Many of the arguments for vaccine hesitancy are based on the assumptions that vaccines are ineffective and involve safety risks [19]. However, the credibility of such claims has been decreased because many of the assumptions presented by anti-vaxxers have been demonstrated to be erroneous. One of the most well-known cases is the refutation of the findings by Andrew Wakefield and 12 of his colleagues [21]. In an article published in Lancet – a prestigious medical journal – they concluded that the measles, mumps and rubella (MMR) vaccine may predispose to behavioural regression and pervasive developmental disorder in children. Despite the small sample size (*n* = 12), the uncontrolled design and the speculative nature of the conclusions, this article received wide publicity. The MMR vaccination rates began to drop because parents were concerned about the risk of autism after vaccination. In 2010, the article was retracted from Lancet as a fraudulent study which is based on the falsification of data [22]. Nevertheless, Wakefield’s findings are still used as an evidence among people who warn about the risks of the COVID-19 vaccines [18].

There is a growing literature examining how vaccine hesitancy is discussed in social media [23–25]. Vaccine hesitancy has gained wider support due to the perceived lack of scientific consensus on vaccine information. This has led to increased reliance on user-generated health information, thus exposing people to vaccine mis/disinformation [26]. It is often spread in emotional narratives about individuals who have allegedly been harmed by vaccinations. In the United States, within 2 days of the first people receiving the Pfizer COVID-19 vaccine, anti-vaccine activists were amplifying stories of allergic reactions or even deaths caused by the vaccine [27]. In such narratives, the risks of vaccine may seem more immediate and tangible as compared with the potential benefits of disease prevention by means of a vaccine [25].

## 3. Conceptual framework

To examine the credibility of COVID vaccine mis/disinformation available in social media forums, this study analyses the messages posted to *VaxxHappened*– a Reddit discussion group which is dedicated to the critique of claims presented by anti-vaxxers. To achieve this, this study makes use of the conceptual framework developed in Savolainen’s [12] investigation of information quality and information credibility. As reviewed above, this study analysed how online discussion participants assess the credibility of information dealing with natural food products and racism. As COVID-19 vaccination similarly represents a controversial topic, Savolainen’s [12] framework was deemed relevant for the present investigation, although it was applied in a modified and condensed form.

Savolainen [12] identified 13 criteria for the assessment of information quality and 13 criteria for the judgement of information credibility. Similar to Savolainen, the present investigation approaches information credibility in terms of the expertise of the author generating messages and submitting posts to online discussion forums. Most importantly, however, and distinct from Savolainen’s study, the category of information quality will not be employed separately. Only the term *information credibility* is referred to while examining the believability of mis/disinformation about the COVID-19 vaccines. This approach is preferred because this study departs from assumption that trustworthiness and expertise are the key aspects of credibility (cf. section 2.1 above). More specifically, it is assumed that expertise is the key aspect of the credibility of the author of the message. However, trustworthiness can also be an aspect of the credibility of the author, particularly if a source (person or document) is judged as a cognitive authority [13]. Importantly, trustworthiness also determines the quality of information content. Information may be of high or low quality, depending on the extent to which information content is judged as being reliable or unreliable, or accurate or inaccurate, for example. Thus, ultimately, (1) credible information content in the sense of trustworthy (reliable, accurate, correct, etc.) information and (2) information quality in the sense of the quality of information content refer to the same phenomena. As there is no need to employ redundant categories, that is, credible information content and information quality, the term information quality will be replaced by the term credibility of information content. Thereby, the trustworthiness of the author of the message, as well as the trustworthiness of the message’s information content can be examined in terms of credibility.

More specifically, as the study focuses on the believability of mis/disinformation, the term *credibility of mis/disinformation content* is used. Second, for clarity, the term information credibility was replaced by the term credibility of the author creating mis/disinformation, more briefly, *credibility of the author*. This term indicates more clearly that the credibility assessment focuses on the believability of the author generating the message’s information content. Moreover, the framework proposed by Savolainen [12] was condensed for the purposes of this study because the preliminary coding of the data downloaded from the Reddit discussion group indicated that criteria such as *variety of information* and *official nature of information* are only marginally relevant for the empirical analysis. It also turned out that some of the criteria defined by Savolainen [12] overlap. For example, *correctness of information* comes close to *objectivity of information* because both categories indicate the degree to which information provides a true and unbiased description of reality. The definition of the relationship of the above concepts is rendered difficult because it is possible that a message that is perceived as unbiased (objective) turns out to be incorrect. Moreover, a biased (opinionated) message may offer correct factual information. However, the preliminary analysis of the empirical material revealed that cases such as these are rare when people judge the credibility of mis/disinformation about COVID-19 vaccines. Therefore, the term *objectivity of information content* is sufficiently nuanced to depict the extent to which information provides true, impartial and unbiased description of reality.

Furthermore, the criterion of *specificity/unspecificity of information* was replaced by the criterion of *accuracy* which was taken from Kim’s [15] investigation. This is because the preliminary coding suggested that accuracy is a more adequate category to depict the details of medical and biological qualities of the COVID-19 vaccines. Finally, Savolainen’s [12] framework was simplified by abandoning the distinction between positive and negative assessment criteria, for example, author’s honesty/dishonesty in argumentation. Although the preliminary coding of the empirical material revealed that almost all credibility judgements tend to be critical, for the sake of simplicity, reverse criteria such as dishonesty and inaccuracy were not used while naming the assessment criteria. Instead, they were labelled in positive or neutral terms, for example, honesty of the author in argumentation and presentation qualities.

**Figure 1.**
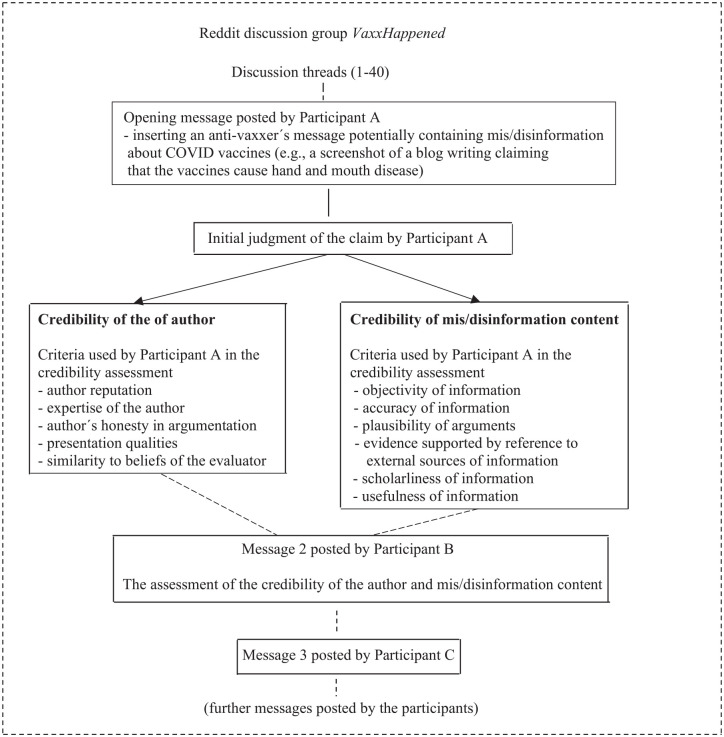
The conceptual framework, modified from Savolainen [12].

As a result of the above modifications, the conceptual framework of the present investigation incorporates five criteria for the assessment of the credibility of the author and six criteria for the judgement of the credibility of mis/disinformation content. The conceptual framework is presented in Figure 1.

Figure 1 illustrates how the credibility assessments are made in the VaxxHappened discussion group. Figure 1 is schematic in that all messages posted to the discussion threads are not necessarily evaluated from the viewpoint of credibility. The discussion is initiated by a message in which the opening poster (Participant A) inserts an anti-vaxxer’s claim about COVID-19 vaccines presented in a blog writing, for example. In the first message, the opening poster then assesses the credibility of the information content generated by the anti-vaxxer, as well as his or her credibility as an author of such information. In the assessment, the opening poster can make use of one or fewer credibility criteria such as the expertise of the author and the objectivity of information to judge whether and in which regard the claim presented by the anti-vaxxer incorporates mis/disinformation. The discussion is continued when other contributors (Participants B, C etc.) make their own credibility assessments. While doing this, they can agree with the initial assessment made by the opening poster or present a different credibility judgement. A similar process occurs when a new discussion thread focusing on another claim about COVID-19 vaccines is initiated.

## 4. Research questions

Drawing on the above framework, this study sought answers to the following questions:

*RQ1.* By drawing on the criteria specified in Figure 1, how do the participants of the VaxxHappened discussion group assess the credibility of authors potentially presenting mis/disinformation about the COVID-19 vaccines?
*RQ2.* By drawing on the criteria specified in Figure 1, how do the participants assess the credibility of the content of such information?

## 5. Empirical data and analysis

The empirical data were gathered from *Reddit*– a social media platform. Reddit advertises itself as ‘the front page of the Internet’ (p. 471) [28] Reddit users (*Redditors*) share news stories and hold conversations within Reddit’s subreddits, that is, subforums. While an account is required for posting to Reddit, content is generally available to be viewed by anyone.

More specifically, the data were downloaded in February 2021 from the subreddit VaxxHappened (https://www.reddit.com/r/vaxxhappened/). This forum is moderated and its slogan boasts to offer ‘$100% true stories from the Anti-vaxx Crowd’. VaxxHappened collects and discusses ‘outrageous and dangerous tales told by dimwitted anti-vaxxers on all forms of media. Post Facebook comments, YouTube videos and gifs’. The group declares to be pro-vaccination because ‘we are civilized and not stupid’ (https://www.reddit.com/r/vaxxhappened/). The discussion topics deal with vaccination against various diseases such as MMR and COVID-19.

To obtain an overall picture of how credibility assessments are made in the above forum, discussion threads focusing on COVID vaccines were read tentatively. The length of such threads varies a lot; some of them contain only a couple of posts while others may attract several hundreds of messages. The sampling criteria required that the thread contains a sufficient number of messages relevant from the perspective of credibility assessment. On the basis of reading 50 threads with the newest updates, a working solution was found: threads containing at least five posts submitted to a discussion thread are sufficient to meet the aforementioned requirement. This is because the participants in all threads, starting from the initial post, were active in presenting comments on the credibility of the author advocating anti-vaxx ideas. In many cases, the first five posts were highly indicative of the main issues reflected in later phases of the discussion. However, it was necessary to limit the number of messages taken into the sample from the longest threads because they tend to repeat what is said in the beginning of the discussion. Therefore, from these threads, only the 200 first messages were taken into the sample. It appeared that the sample containing threads with 5–200 messages is sufficient to allow a detailed analysis of the credibility assessments made by the Redditors. Finally, 40 threads initiated within the period of 1 January to 7 February 2021 were chosen for the sample. Since the study does not aim at producing statistically representative generalisations, this sample appeared to be sufficient for the needs of the present investigation. It became evident that the inclusion of additional threads would not have essentially changed the quantitative and qualitative picture of how the Redditors assess the credibility of COVID-19 vaccine mis/disinformation. Said otherwise, the data became saturated because after the preliminary analysis of 30 discussion threads it appeared that most of the credibility judgements presented by the online participants are based on similar arguments. However, to ensure further saturation, the preliminary analysis was continued by scrutinising 10 additional threads.

The 40 threads contained in total 1877 messages. As illustrated in Figure 1 above, the opening message of each thread incorporates a description of mis/disinformation created by an anti-vaxxer; usually, such information was presented by inserting a screenshot from the message potentially containing mis/disinformation. Anti-vaxxers claimed, for example, that ‘the Phiz/a vaxx killed 33 people’ (Thread 2), COVID vaccines will be ‘permanently altering RNA, one arm at a time’ (Thread 19) and ‘Doctors around the world (give) dire warning: don’t get the COVID vaccine!’ (Thread 40).

Altogether 1005 individual Redditors contributed to the 40 discussion threads. Of the contributors, 653, that is, 65% were occasional participants in that they wrote only one message. In contrast, there was a handful of a really active Redditors; of them, the most frequent participant posted no less than 27 messages to diverse discussion threads. All in all, the topics related to COVID vaccines attracted a high number of participants. However, only a few appeared to be continually interested in vaccinations issues.

The sample of 40 threads was downloaded in a separate file and coded by making use of the categories specified in Table 1.

**Table 1.** Criteria used in the credibility assessment, modified from Savolainen [12] and Kim [15].

Criterion	Short definition and examples from coded material
Credibility of the author	
Author reputation	The extent to which the author is generally evaluated positively or negatively in a community (‘Catie is seriously messed up. At times, I feel sorry that she got herself lost in a bunch of baloney’, Thread 21).
Expertise of the author	The extent to which the author is considered as competent in a specific area (‘This cannot be real. They cannot be this ignorant’, Thread 33).
Honesty in argumentation	The extent to which the author is able to consider an issue in a sincere way (‘Their willful ignorance is just astounding’, Thread 1).
Presentation qualities	The extent to which the author is able to communicate his or her ideas clearly and using appropriate language (‘When I see someone typing about, mumma, pappa, yr, Phiz/a etc. I know that whatever that person is trying to communicate is mostly bs’, Thread 2).
Similarity to receiver beliefs	The degree to which the ideas presented by the author are found as acceptable due to compatibility with one’s own views (‘They are more likely to listen to us than strangers, aka trained scientists, so although it sucks, the arguing helps’, Thread 3).
Credibility of the message’s information content	
Objectivity of information	The extent to which information provides true, impartial and unbiased description of reality (‘mRNA does not rewrite and eventually leaves when the cell dies when the white blood cell dies which it only interacts with a select few’, Thread 28).
Accuracy of information	The extent to which information provides an exact description of reality (‘You are right, they don’t contain foetal cells, but it is not entirely accurate to say the Pfizer vaccine has nothing to do with foetal cell lines’ Thread 11).
Plausibility of arguments	The extent to which information is based on valid and logical argumentation (‘Anti-vaxxers have a very twisted logic: Person dies Covid positive => They died with Covid, not from it. Person dies after receiving the vaccine => They died from the vaccine. As others already pointed out, this is a blatant misrepresentation as usual’, Thread 2).
Evidence supported by reference to external sources of information	The extent to which information is supported by relevant documents used as evidence reference to external sources of information (‘This is the second post about the video, so here is the link. The anti-vaxxer’s claims are just ridiculous: https://youtu.be/aRWHMfoNSJs’, Thread 14).
Scholarliness of information	The extent to which information is based on the findings of scientific research (‘They are calling the wide distribution of the vaccine to the public a “study,” and saying they are in the control group meaning they will not be taking it. They are anti-vaxx’. Thread 10).
Usefulness of information	The extent to which information is considered as helpful to meet the need of a person or a group (‘Makes me angry that anti-vaccers and conspiracy theorists will use and twist these kind of stories to manipulate and scare people who don’t know better or how to do or understand research’, Thread 2).

The coding was an iterative process in which the data were scrutinised several times by the author. The pre-defined categories specified in Table 1 were then used to code all the data – while still allowing new codes to emerge. However, all credibility assessments fit into the existing categories defined in Table 1 and no new categories were needed to cover the data. The 1877 posts were assigned with altogether 787 codes. They always dealt with the credibility assessment of the claims presented by the anti-vaxxers. The number of codes is lower than the total number of posts because only 603 posts out of 1887 contained explicit credibility assessments while the rest (1284 posts) did not incorporate such assessments. Thus, the share of posts containing credibility judgements was 32%. On average, the number of codes per coded post was 1.3 and the maximum number of codes per post was 3. A message was coded only once for a criterion category once it was identified for the first time in the message. In long posts in particular, it was not unusual that the same criterion was identified in several segments of the same post. In these cases, once a message was coded for a criterion category, other instances were simply ignored. However, a post could be assigned with several criteria of author credibility and/or the credibility of information content, for example, author reputation and accuracy of information.

The internal reliability of the coding was improved in that the coding categories specified in Table 1 are built on the solid foundation of research on information credibility [12,15]. To strengthen the reliability of the coding, only explicit judgements concerning credibility assessment were coded using the categories specified in Table 1. Moreover, the initial coding was refined by repeated reading of the data, that is 40 threads with 5–200 posts. Miles and Huberman [29] noted that check-coding the same data is very useful for the lone researcher and that code-recode consistencies should be at least 90%. To achieve this, eight iterations were executed so that in the final phase, the codes assigned to individual categories, most notably ‘accuracy of information’ and ‘objectivity of information’, were changed to better capture the content of a credibility judgement. Following this advice, the coding was refined until it was found that the codes appropriately describe the data and that there are no anomalies.

In order to examine the relative share of the credibility criteria, percentage distribution was calculated for individual criteria used in the assessment of author credibility and the credibility of information content. To this end, the number of codes assigned to a category, for example, author reputation (*n* = 157) was divided by the total number of the codes, that is, 787. Second and more importantly, the data were scrutinised by means of qualitative content analysis. To achieve this, the constant comparative method was used to capture the variety of articulations of the Redditors’ credibility assessments [30]. More specifically, the Redditors’ comments on the credibility of mis/disinformation were systematically compared per individual criteria. In this way, it was possible to identify similarities and differences in the ways in which the Redditors assessed, for example, the expertise of the author or the objectivity of information about the side effects of COVID-19 vaccines.

The reporting of the qualitative findings incorporates an ethical issue because they are illustrated by quotations taken from the Redditors’ messages. Since the messages posted to the VaxxHappened group are freely accessible to all readers, these messages can be seen as contributions which are intended to elicit public interest in COVID-19 vaccination issues. Due to their public nature, the messages mailed to online forums may also be utilised for research purposes, provided that the identity of an individual contributor is sufficiently protected. To achieve this, participants were identified by technical codes. For example, P-766 refers to a contributor who appears in the 766th place in the alphabetical list of 1005 Redditors. Moreover, individual threads were referred to using a technical code. For example, T-24 refers to a message that appeared in Thread 24. Second, all information about the submission dates for messages was deleted from the quotations. This procedure makes it more unlikely that an individual message and its author could be identified from the VaxxHappened discussion threads.

## 6. Findings

### 6.1. Quantitative overview

Of the codes assigned to credibility assessments, a slight majority (53.9%) dealt with the credibility of the authors presenting claims about the COVID-19 vaccines, while the share of codes assigned to the credibility of the information content was somewhat lower, that is, 46%. The percentage distribution of the codes is presented in Table 2.

**Table 2.** Codes assigned to the credibility assessments (*n* = 787).

Criterion	%	*n*
Credibility of the author		
Author reputation	20.0	157
Honesty in argumentation	14.2	113
Expertise of the author	13.6	107
Presentation qualities	4.7	37
Similarity of beliefs	1.4	11
Subtotal	53.9	425
Credibility of the information content		
Objectivity of information	21.7	171
Plausibility of arguments	11.8	93
Evidence (reference to external sources)	6.7	53
Accuracy of information	2.7	21
Usefulness of information	1.8	14
Scholarliness of information	1.3	10
Subtotal	46.0	362
Total	99.9 (due to rounding)	787

As to the credibility of the author, the most frequent assessments focused on his or her reputation, honesty in argumentation and perceived expertise. To compare, presentation qualities and similarity of beliefs occupied only a marginal role in the credibility judgements. Regarding the assessment of the information content created by anti-vaxxers, the Redditors most frequently drew attention to the objectivity of information and the extent to which the arguments presented by anti-vaxxers are plausible and logical. In fewer cases, Redditors assessed whether information is supported by evidence with references to external sources of information. All in all, the quantitative overview suggests that the credibility assessment of claims potentially containing mis/disinformation is based on the use of a few key criteria, most notably author reputation, his or her honesty and expertise, as well as the objectivity of information and the plausibility of arguments.

### 6.2. Qualitative features of credibility assessments

#### 6.2.1. Credibility of the author

As a point of departure, Redditors often evaluated the reputation of the anti-vaxxer presenting claims about COVID-19 vaccines. Unsurprisingly, the tone of such assessments was negative, thus suggesting that pro-vaxx and anti-vaxx people belong to the opposite camps, with incompatible values and deep mutual distrust. The assessment of the author reputation was most explicit in cases in which the advocates of vaccine hesitancy were identified by name. Well-known anti-vaxxers such as Andrew Wakefield and Carrie Madej attracted Redditors’ particular attention. Anti-vaxxers’ reputation was put in a dubious light by ridiculing and disparaging them. Ad hominem attacks were usual. In the harshest comments, anti-vaxxers were labelled as ‘idiots’ (P-862-T-3), ‘morons’ (P-20-T-3), ‘liars’ (P-462-T-38) and ‘quacks’ (P-790-T-40):

Oh, looky look, it is Carrie Madej, a notorious DO (not an MD) who thinks vaccines will put us into the matrix. (P-385-T-40)

I usually call him Andrew Fakefield. (P-240-T-38)

Another factor significantly affecting the reputation of the author is his or her professional credentials. Almost without exceptions they were assessed negatively by emphasising, for example, that the anti-vaxxer is morally dubious or that his medical licence is suspended:

He is both scientifically and morally corrupt. Now, it appears he is an expert on the COVID-19 vaccines. Once you throw away your livelihood to make a quick buck, grifters got to grift. It is all he is good at. (P-577-T-38)

A significant aspect in the evaluation of the author credibility is his or her perceived expertise, that is, the extent to which the author is considered as competent in a specific area. Similar to the assessment of author reputation, Redditors took a very critical stance on the anti-vaxxers’ competence, thus suggesting that their claims about COVID-19 vaccines incorporate mis/disinformation, instead of valid facts and reasoning:

They don’t even understand what a virus is, so getting them to understand things like RNA and DNA is a total lost cause. (P-40-T-22)

From the Redditors’ point of view, the incompetency of anti-vaxxers manifests itself most clearly in the erroneous conceptions of the biological nature of the COVID-19 vaccines and the ways they work. One of the indicators of incompetency among anti-vaxxers is the speculative assumption that along the COVID-19 vaccine, the vaccinated person actually gets the coronavirus. Anti-vaxxers believe that assumptions of this kind can be proved to be true by doing ‘your own research’, instead of relying on the findings of scientific vaccine research:

That is sadly how a lot of conspiracy theories come to be. People see a fact that does not (fit) 100% for into their world view and instead of asking professionals, people who have studied this for years, they go off and make up their own theory and declare it as fact. (P-255-T-13)

The perceptions of author credibility are also affected by the extent to which the author is able to consider an issue in a sincere way. One of the Redditors’ main arguments is that anti-vaxxers’ ignorance about the nature and effects of COVID-19 vaccines is ‘wilful’. As one of the Redditors put it, ‘this dishonesty is just astounding’ (P-240-T-1). Moreover, as judged by the Redditors, anti-vaxxers’ credibility as creators of believable information is undermined by the fact that they present themselves as experts, pretending that they possess relevant knowledge about the COVID-19 vaccines. However, this impression may be misleading because the evidence they draw on vaccination-related studies tend to be biased. From the pro-vaxxers’s point of view, author credibility is also weakened because anti-vaxxers tend to appeal on people’s emotions such as fear and anxiety rather than presenting facts about the vaccines. As critiqued by a Redditor (P-275-T-23), dishonesty in argumentation manifests itself in ‘emotional fear-based theatrics to win hearts and minds’. ‘Theatrics’ of this kind may appear dramatically in videos advocating vaccine refusal:

She (=Carrie Madej) then tells us that she knows this is a lot of information to digest, which she apparently has not been able to do because she has to read every word of this diatribe. As a finale, this specimen gets all emotional, and believe it or not, starts to cry at the prospect of the world being exposed to the vaccine. The barrage of crocodile tears loaded with putrid pseudoscience is enough to make anyone with a modest scientific background cry. (P-385-T-40)

The biased approach indicative of dishonesty in argumentation became manifest in a case in which anti-vaxxers claimed that in January 2021, after receiving the COVID-19 vaccine, 33 elderly people died in Norway. Anti-vaxxers speculated whether the death cases would have been caused by the vaccine. As argued by the Redditors, inferences such as these offer disinformation which is based on intentional misinterpretation of the Norwegian case. To this end, disproportionately strong attention was devoted to a relatively small number of deaths, instead of relating them to a broader picture of the pandemic and the scale of vaccination worldwide:

400K Covid deaths. It is just the flu. Only a 2% mortality rate. 33 vaccine deaths. Far too many. (P-71-T-2)

Dishonesty in argumentation also manifests itself in the exaggeration of the side effects of COVID-19 vaccines. One of the forums used for intentional spreading of biased information is *Vaccine Adverse Event Reporting System* (VAERS). It relies on individuals’ self-reported data about their vaccination experiences:

Anti-vaxxers have used VAERS to make false claims for years. Yes, there are vaccine reactions and yes, people can be allergic. But anti-vaxxers misrepresent side effects as adverse reaction. (P-247-T-24)

Author credibility is often affected by the ways in which information is presented, that is, the extent to which the author is able to communicate his or her ideas clearly and using appropriate language. Therefore, the writing skills and the command of relevant terminology are particularly important. The Redditors were keen to identify grammatical errors and terminological problems in the claims presented by anti-vaxxers. Often, anti-vaxxers’ communication abilities were disparaged and derided:

When I see someone typing about mumma, pappa, yr, Phiz/a etc., I know that whatever that person is trying to communicate is mostly bs. (P-163-T-2)

Finally, the assessment of author credibility may be affected by the degree to which the ideas presented by the author are found as acceptable due to compatibility with one’s own views. As a whole, this criterion appeared to be quite marginal. However, there were a few examples which were mainly indicative of how the Redditors reflected the anti-vaxxers’ claims in relation to the ideas of vaccine hesitancy. Again, the Redditors’ comments indicated the existence of opposite beliefs regarding the pros and cons of the COVID-19 vaccines in family debates, for example:

They have tried to convince me to see if I could sign a form for nursing school saying that it was against my religion to get vaccinated. It is not, and I wanted to be vaccinated since I would be working with sick people at the hospital. (P-78-T-3)

#### 6.2.2. The credibility of mis/disinformation content

As Table 2 above indicated, the most frequently mentioned criterion in the assessment of the credibility of information content was objectivity of information, that is, the extent to which information provides true, impartial and unbiased description of reality. Overall, Redditors appeared to be highly doubtful about the objectivity of information offered by anti-vaxxers because they were associated with the intentional distribution of untrue messages. In some cases, the claims presented by the anti-vaxxers were bluntly labelled as ‘QAnonsense’ (P-664-T-11), linked with the conspiracy theories advocated by the *QAnon* movement. The credibility of vaccine information distributed by anti-vaxxers was also doubted because they presented erroneous assumptions such as that atoms would have DNA. The question of the objectivity of information content was also assessed while commenting on the anti-vaxxers’ claims that COVID-19 vaccines would mutate people’s DNA. To refute such assumptions, the Redditors made an attempt to correct mis/disinformation by introducing facts that are based on scientific research:

Madej starts out by describing correctly the role that DNA plays as the blueprint for virtually everything that happens in our body, and then proceeds to jump off the springboard directly into an empty pool. The mRNA vaccines, she says, are going to convert us into genetically modified organisms by altering our DNA. This is absolute nonsense. mRNA does not get incorporated into DNA. All it does is code for the production of the virus’ spike protein which in turn stimulates antibody production. (P-385-T-40)

The above criticism suggests that the claims presented by anti-vaxxers suffer from unspecific assumptions. However, accuracy of information is an important factor affecting the credibility of information content, that is, the extent to which information provides an exact description of reality. In this regard, Redditors identified a number of loose assumptions presented by anti-vaxxers:

For the last time: the Pfizer (and Moderna) vaccine does not contain any fetal cells or fetal cell lines! The AstraZeneca one does. Of all vaccines anti-abortion people could get behind, the mRNA vaccines would be it. They use a completely different technology. Nothing to do with aborted fetal cells at all. (P-346-T-11)

Similar to objectivity of information, plausibility of arguments was one of the most frequently used criteria in the assessment of the credibility of information content. Plausibility of arguments indicates the extent to which information presented in a message is based on valid and logical argumentation. As suggested above, the claims presented by anti-vaxxers tend to be biased in that they intentionally emphasise certain issues while wilfully ignoring others because they do not fit to their agenda. More specifically, there appeared to a number of inconsistencies and logical fallacies in the ways in which the anti-vaxxers grounded their claims. Anti-vaxxers were also ridiculed for their ways to draw conclusions from the apparent similarity of diverse phenomena. This is indicative of lazy thinking:

Anti-vaxxers have a very twisted logic: Person dies Covid positive => They died with Covid, not from it. Person dies after receiving the vaccine => They died from the vaccine. This is a blatant misrepresentation as usual. (P-240-T-2)

Interestingly, in a few cases, the Redditors admitted that some of the claims about the safety risks are partially plausible because the long-time effects of the COVID-19 vaccines are still uncertain:

Counterpoint, we don’t know (neither do doctors nor specialists) the adverse side effects that could develop over time. We don’t know (assuming there is some sort of side effect) if it will affect a minority of people that got vaccinated, or a majority. We don’t know, and that’s why I don’t trust it fully, there are too many unknowns, unlike with vaccines that have been used for a long time, we know the negative effects, and we have a fairly good understanding of the chance. (P-256-T-40)

Many of the critical assessments of the dubious credibility of information content created by anti-vaxxers was based on the lack of sufficient evidence supported by references to external sources such as relevant documents. One of the recurrent slogans presented by the Redditors was ‘Link or it did not happen’ (P-635-T-2):

But what really got to me was his complete lack of sources. Like, he did not even try to use some news source. He just shrugged! I have still no idea where he got 40,000 from. (P-543-T-25)

Similarly, criticism was directed to the poor quality of information sources such as tweets, YouTube videos and Facebook messages. The anti-vaxxers’ predilection to draw on undocumented personal sources, for example, ‘I heard someone on Facebook say’ was severely criticised (P-621-T-33). Closely related to the lack of reliable evidence, the credibility of information content is affected by the scholarliness of information, that is, the extent to which information is based on the findings of scientific research. This is particularly important in the case of vaccination issues because they ultimately draw on biological and medical knowledge. As noted above, however, most of the evidence cited by anti-vaxxers tends to originate from non-scholarly sources such as blog writings and self-reported data submitted to the VAERS system. This bias was also reflected in the Redditors’ comments. Many of them were sarcastic and ridiculed how anti-vaxxers make use of information sources to support their claims:

Unfortunately, for a lot of people, Twitter posts and screenshots are all part of their ‘research’. (P-473-T-4)

Finally, the credibility of information source content can be affected by the assessment of the usefulness of information, that is, the extent to which information is considered as helpful to meet the need of a person or a group. From the viewpoint of the Redditors, the advice offered by the anti-vaxxers may have detrimental health consequences for people who objectively need the COVID-19 vaccine, due to the chronic diseases such as asthma and diabetes:

That is what makes it so dangerous. There are little nuggets of truth which make them seem almost a bit credible to a layperson that is on the fence. (P-142-T-29)

## 7. Discussion

This study contributed to empirical research on information credibility by analysing how the believability of mis/disinformation content is judged in online discussion. It was also examined how the credibility of authors creating such information is assessed in online discourse. The main findings are summarised in Table 3.

**Table 3.** Summary of the main findings.

	Main approaches used in credibility assessment
• Credibility of the author (RQ1)	
Author reputation	Disparaging the authors by ad hominem attacks. Putting the authors’ professional credentials in a dubious light.
Author’s expertise	Labelling the authors of as incompetent people having inadequate and biased knowledge about the COVID-19 vaccines. Identifying concrete examples of erroneous assumptions of the nature of vaccines.
Author’s honesty in argumentation	Accusing the authors for wilful ignorance of the nature and effects of the COVID-19 vaccines. Offering concrete examples of how the authors exaggerate the risks of the vaccine.
Presentation qualities	Ridiculing grammatical errors and awkward expressions. Criticising the vague (pseudo-scientific) terminology used by the authors.
Similarity of beliefs	Providing concrete examples of dissimilarity of vaccination-related beliefs and values.
• Credibility of mis/disinformation content (RQ2)	
Objectivity of information	Asserting that the information distributed by anti-vaxxers is untrue and biased. Refuting the assumptions of anti-vaxxers by presenting factual knowledge, based on the findings of scientific research.
Accuracy of information	Identifying factual errors in the vaccine-related claims presented by anti-vaxxers. Correcting faulty assumptions by presenting facts obtained from external sources such as statistics.
Plausibility of arguments	Identifying cases of biased argumentation. Identifying logical fallacies and examples of lazy thinking.
Evidence supported by references	Critiquing for the lack of references made to reliable sources. Ridiculing the author’s confidence on non-authoritative sources such as Facebook pages and hearsay of other people.
Scholarliness of information	Critiquing the intentional ignorance of information sources offering scientific information about the COVID-19 vaccines.
Usefulness of information	Asserting that mis/disinformation offered by anti-vaxxers is potentially dangerous for people with chronic condition in particular.

Overall, the study revealed that the credibility of anti-vaxxers as creators of vaccine-related information is very low in the eyes of pro-vaxx online discussion contributors. Anti-vaxxers were characterised as people with poor reputation because they lack adequate knowledge of the nature and effects of the COVID-19 vaccines. Anti-vaxxers were found dishonest because they deliberately draw on false or biased evidence in order to exaggerate the risks of COVID-19 vaccines. Moreover, anti-vaxxers were ridiculed for their inability to express their claims logically and clearly. As to the credibility of the information content, the Redditors’ assessments were similarly negative. They asserted that the anti-vaxxers’ claims about COVID-19 vaccines are false and inaccurate. Anti-vaxxers’ conclusions are based on selective use of evidence supporting their agenda. The credibility of vaccine-related information is also decreased by the ignorance of scholarly information sources. Finally, from the Redditors’ point of view, the claims presented by the anti-vaxxers tend to have low use value for those considering vaccination decision; in the worst cases, vaccine refusal advocated by the anti-vaxxers can endanger the health of people with chronic condition.

The findings support the observations of earlier studies on credibility assessment occurring in online discussion forums. Similar to Kim [15] and Savolainen [12], it was found that the judgements concerning the person’s reputation, expertise and presentation qualities are key criteria used in the assessment of the author’s credibility. The results of this study also confirm the conclusions drawn in the above investigations in that objectivity and accuracy of information, as well as logical argumentation are highly important in the evaluation of the credibility of the message’s information content. Therefore, one of the main conclusions of the present investigation is that ultimately, the assessment of the credibility of truthful information and mis/disinformation is based on similar criteria, although in the latter case they are interpreted in a ‘reverse’ way so that the main attention is devoted to the lack of positive qualities, for example, incompetency of the author or inaccuracy of information.

The findings also lend support to the conclusions drawn by Yin and Zhang [3]. Their study on the ways in which users evaluate microblog information credibility revealed that argument quality (related to the message content) is a factor that people tend to take for granted; poor argument quality is simply unacceptable. However, information credibility can be best increased by enhancing the credibility of author: the more credible the author, the more users rely on information available in a blog. Similarly, the findings of this study suggest that if the credibility of the author is seriously doubted due to the person’s poor reputation, dishonesty or incompetency, it is difficult or even impossible to rely on any information content created by that person. Therefore, due to this labelling effect, author credibility is crucially important for the credibility judgement as a whole.

Interestingly, in their negativity, credibility assessments made by the Redditors were highly similar. This is probably due to the strong pre-message expectancies of the nature of anti-vaxxer’s claims; they should be put in a dubious light because they have an obvious persuasive intent [14]. In fact, there was only a couple of cases in which the Redditors’ comments offered partial support for the anti-vaxxers’ claims dealing with the uncertainty of the long-term effects of the COVID-19 vaccines. The broad consensus among the Redditors suggests that in online communities, credibility assessment is not necessarily based on the solitary action performed by an individual; rather, such judgements can be understood as something that is constructed in relation to a specific social setting such a group of like-minded individuals [31]. Therefore, pro-vaxx Redditors are affected by the social endorsement on credibility perceptions [32]. The endorsement heuristic – also known as the ‘bandwagon effect’– implies that one tends to agree with an interpretation that has been endorsed by many others. However, the mechanism of social endorsement may also have a ‘narrowing effect’ on credibility assessments because it is expected that such judgements should support the agenda of the online community, either pro- or anti-vaccination.

## 8. Conclusion

As the ‘post-truth’ era is increasingly characterised by the ‘swirling cacophony of competing viewpoints, perspectives, agendas, and facts’ (p. 183) [5], the assessment of claims potentially incorporating dis/misinformation is highly important in times of pandemics such as COVID-19. However, it is not reasonable to label outright all messages available in the social media as mis/disinformation. It is more important to consider in what regard and by which criteria would such messages be judged as information of this kind. The findings of this study highlight that in such assessments, the criteria pertaining to the author’s reputation, expertise and honesty are particularly significant, similar to the objectivity of information content. As this study focused on credibility assessment made by participants oriented by strong pre-message expectancies, the findings cannot be generalised to concern online discourse on vaccination in all social media forums. More research is required to capture a broader picture of the ways in which people accept or refute messages potentially containing mis/disinformation, as opposite to correct and objective information. For example, Ruokolainen and Widén [33] have recently demonstrated that the ways in which asylum seekers perceive messages as accurate, that is, correct or objective, misinformation or disinformation are affected by social, cultural and situational factors functioning as ‘filters’ that are used in the interpretation of the message content. Therefore, accurate information, misinformation and disinformation form a continuum where the borders between the above categories are not always clear. Further studies delving deeper into the specific features of mis/disinformation would also deepen our understanding about the criteria by which people sometimes find such information believable as a basis of health-related decision-making.
